# Histological Examination in Obtaining a Diagnosis in Patients with Lymphadenopathy in Lima, Peru

**DOI:** 10.4269/ajtmh.16-0961

**Published:** 2017-08-21

**Authors:** Daniela E. Kirwan, Cesar Ugarte-Gil, Robert H. Gilman, Syed M. Hasan Rizvi, Gustavo Cerrillo, Jaime Cok, Eduardo Ticona, José Luis Cabrera, Eduardo D. Matos, Carlton A. Evans, David A. J. Moore, Jon S. Friedland, The Lymph Node Tuberculosis (LNTB) Working Group

**Affiliations:** 1 Department of Medical Microbiology, St. George’s Hospital, London, United Kingdom;; 2 Department of Infectious Diseases and Immunity, Imperial College London, London, United Kingdom;; 3 Wellcome Trust Centre for Global Health Research, Imperial College London, United Kingdom;; 4 Instituto de Medicina Tropical Alexander von Humboldt, Universidad Peruana Cayetano Heredia, Lima, Peru;; 5 Facultad de Medicina Alberto Hurtado, Universidad Peruana Cayetano Heredia, Lima, Peru;; 6 Department of International Health, Johns Hopkins University, Baltimore, Maryland;; 7 Laboratorio de Investigación en Enfermedades Infecciosas, Universidad Peruana Cayetano Heredia, Lima, Peru;; 8 Department of Cellular Pathology, Barts Health NHS Trust, London, United Kingdom;; 9 Infectious Diseases and Tropical Medicine Unit, Hospital Nacional Dos de Mayo, Lima, Peru;; 10 Department of Pathology, Hospital Nacional Cayetano Heredia, Lima, Peru;; 11 Infectious Diseases and Tropical Medicine Unit, Hospital Nacional Dos De Mayo, Lima, Peru;; 12 Department of Medicine, Universidad de San Marcos, Lima, Peru;; 13 Department of Pulmonology, Hospital Daniel Alcides Carrión, Callao, Peru;; 14 Department of Infectious Diseases, Hospital Nacional Arzobispo Loayza, Lima, Peru;; 15 Innovation for Health and Development, Laboratory of Research and Development (IFHAD), Universidad Peruana Cayetano Heredia, Lima, Peru;; 16 TB Centre, London School of Hygiene and Tropical Medicine, London, United Kingdom

## Abstract

The differential diagnosis for lymphadenopathy is wide and clinical presentations overlap, making obtaining an accurate diagnosis challenging. We sought to characterize the clinical and radiological characteristics, histological findings, and diagnoses for a cohort of patients with lymphadenopathy of unknown etiology. 121 Peruvian adults with lymphadenopathy underwent lymph node biopsy for microbiological and histopathological evaluation. Mean patient age was 41 years (Interquartile Range 26–52), 56% were males, and 39% were HIV positive. Patients reported fever (31%), weight loss (23%), and headache (22%); HIV infection was associated with fever (*P* < 0.05) and gastrointestinal symptoms (*P* < 0.05). Abnormalities were reported in 40% of chest X-rays (*N* = 101). Physicians suspected TB in 92 patients (76%), lymphoma in 19 patients (16%), and other malignancy in seven patients (5.8%). Histological diagnoses (*N* = 117) included tuberculosis (34%), hyperplasia (27%), lymphoma (13%), and nonlymphoma malignancy (14%). Hyperplasia was more common (*P* < 0.001) and lymphoma less common (*P* = 0.005) among HIV-positive than HIV-negative patients. There was a trend toward reduced frequency of caseous necrosis in samples from HIV-positive than HIV-negative TB patients (67 versus 93%, *P* = 0.055). The spectrum of diagnoses was broad, and clinical and radiological features correlated poorly with diagnosis. On the basis of clinical features, physicians over-diagnosed TB, and under-diagnosed malignancy. Although this may not be inappropriate in resource-limited settings where TB is the most frequent easily treatable cause of lymphadenopathy, diagnostic delays can be detrimental to patients with malignancy. It is important that patients with lymphadenopathy undergo a full diagnostic work-up including sampling for histological evaluation to obtain an accurate diagnosis.

## BACKGROUND

The differential diagnosis for lymphadenopathy is wide and includes infectious, immunological and metabolic disorders, and primary or secondary neoplasms.^1,2^ In the developed world, common infectious causes predominate including upper respiratory tract infections, Epstein–Barr Virus, and cytomegalovirus, whereas in resource-poor settings other infections such as tuberculosis (TB), toxoplasmosis, HIV seroconversion, leishmaniasis, and fungal infections may be important causes.^3^ Malignant causes such as lymphoma and leukemia are less frequent but correct and prompt diagnosis has prognostic and therapeutic implications. Rarer disorders such as systemic lupus erythematosus and storage diseases are also causes of lymphadenopathy. HIV-infected patients are particularly susceptible to developing hematological malignancies and to acquiring bacterial, fungal, parasitic, and viral infections.

The pathophysiology, treatment, and prognosis of the above conditions differ markedly, and it is therefore crucial to reach a correct diagnosis for each patient. Diagnostic delays can be detrimental to patients.^4^ Few reviews of the clinical and etiological spectrum of lymphadenopathy in resource poor countries exist in the recent literature. Herein, we report the diagnoses and clinical features of a cohort of patients being investigated for lymphadenopathy in Lima, Peru, and demonstrate the importance of histopathological characterization of disease.

## MATERIALS AND METHODS

Patients were prospectively recruited over a 14-month period from the infectious diseases, head and neck surgery, and general medical and surgical departments of three public hospitals in Lima, Peru: the Hospital Nacional Dos De Mayo, Hospital Nacional Daniel Alcides Carrión, and Hospital Nacional Arzobispo Loayza. Lymphadenopathy was defined as enlargement of one or more lymph nodes. All adult patients with lymphadenopathy of unknown cause and in whom diagnostic lymph node tissue sampling was indicated were eligible for the study. This was carried out as part of a prospective evaluation for the accuracy of the Microscopic-Observation Drug-Susceptibility assay (MODS)^5^ for the diagnosis of lymph node TB, reported elsewhere.^6^ Ethical approval was obtained from the Institutional Ethics Committee of the Universidad Peruana Cayetano Heredia, Asociación Benéfica PRISMA, and each study site’s local ethics committee. Written informed consent was obtained from all patients. Demographic and clinical data were collected from patients using a standardized form. Before the biopsy, the patients’ physician was asked to predict the most likely diagnosis based on available clinical information. Tissue sampling procedures and clinical management were undertaken by the hospital clinical staff with no input from the study team. Where HIV status was not known, an HIV test was offered.

After sampling, tissue was divided into three parts and underwent histopathological evaluation, microbiological testing for TB, and standard fungal and bacterial culture. For histopathological assessment, samples were immediately placed in formaldehyde and transported to the hospital pathology laboratory where the samples were sealed in paraffin blocks and reported routinely. After completion of enrolment, the paraffin blocks were retrieved and slides for this study were fixed and stained with hematoxylin–eosin, Ziehl–Neelsen, and Periodic acid–Schiff (PAS) stains, and analyzed by three pathologists who were blinded to clinical data to minimize bias. A standardized reporting form was completed on which the presence or absence of acid-fast bacilli (AFB), granulomas, and caseating necrosis were recorded, and a diagnosis was given based on overall assessment. A histological diagnosis was assigned to the patient when there was concordance between two or more pathologists. Microbiological testing for TB comprised Auramine microscopy, Lowenstein–Jensen culture, and the MODS assay, and microbiological criteria for a TB diagnosis were positivity according to one or more of these tests. A diagnosis of TB was assigned to patients in whom histological and/or microbiological criteria were met.

Data were entered into Excel spreadsheets and analyzed using Stata version 12 (StataCorp, College Station, TX). Comparisons were made using Fisher’s exact test for nominal data and the Mann–Whitney *U* test for continuous data. A *P* < 0.05 was considered significant. Agreement between the pathologists was assessed using Cohen’s Kappa coefficient for multiple ratings (κ);κ ≥ 0.61 indicates substantial agreement.^7^

## RESULTS

One hundred and thirty-two patients agreed to participate in the study and underwent biopsy. Paraffin blocks were retrieved for full histopathological evaluation for 121/132 (92%) patients. The 11/132 (8.3%) for whom this had not been possible were excluded from all analyses. There was no significant difference in age, sex, frequency of HIV positivity, or rate of previous TB between patients who were included in and those who were excluded from analysis.

Patient demographics are presented in Table 1. 55% of the patients were male, and median age was 41 years (interquartile range [IQR] 26–52 years). HIV status was available for 109 patients, of whom 47 (43%) were HIV-positive. Median CD4 count, which was available for 31 patients, was 156 cells/mm^3^ (IQR 41–277). Seventeen HIV-positive patients (36%) were taking antiretroviral drugs at the time of biopsy. HIV-positive patients were more likely than HIV-negative patients to be male (75% versus 43%, *P* = 0.001) and were younger (33 versus 45 years, *P* = 0.006).

**Table 1:** Patient demographics, clinical characteristics, and radiological features

	HIV positive (*N* = 47)	HIV negative (*N* = 74)	*P* value	Total (*N* = 121)
Patient demographics				
Females (%)*	12 (26)	42 (57)	0.001	54 (45)
Age, median years (IQR)†	33 (26–43)	45 (26–62)	0.0063	41 (26–52)
CD4 count, median cells/mm^3^ (IQR)†	156 (41–277)	N/A	N/A	156 (41–277)
HIV-positive patients receiving antiretroviral therapy at time of biopsy (%)*	17/47 (36)	0	1.0	17/47 (36)
Previous TB (%)*	10 (21.3)	11 (14.9)	0.46	21 (17)
Duration of symptoms, months. Median (IQR)†	3 (1–9)	4 (1.5–12)	0.24	3 (1–12)
Symptom				
Fever (%)*	20 (43)	18 (24)	0.045	38 (31)
Weight loss (%)*	13 (28)	15 (20)	0.38	28 (23)
Headache (%)*	11 (23)	15 (20)	0.82	26 (22)
Cough (%)*	11 (23)	11 (15)	0.33	22 (18)
Local pain or tenderness (%)*	5 (11)	15 (20)	0.21	20 (17)
Malaise (%)*	10 (21)	10 (14)	0.32	20 (17)
Appetite loss (%)*	6 (13)	5 (6.8)	0.36	11 (9.1)
Gastrointestinal symptoms (%)*	7 (15)	3 (4,1)	0.045	10 (8.3)
Neck pain (%)*	4 (8.5)	6 (13)	1.000	10 (8.3)
Dyspnea (%)*	6 (13)	2 (4.2)	0.055	8 (6.6)
Asymptomatic (%)*	3 (6.4)	6 (13)	1.0	9 (7.4)
Radiological findings				
CXR normal (%)*	21 (45)	40 (54)	0.35	61 (50)
CXR abnormal (%)*	20 (43)	20 (27)	0.11	40 (33)
Pleural effusion(s) (%)*	4 (8.5)	8 (11)	0.76	12 (9.9)
Pulmonary infiltrates and/or consolidation (%)*	4 (8.5)	7 (9.5)	1.0	11 (9.1)
Hilar and/or paratracheal adenopathy (%)*	2 (4.3)	6 (8.1)	0.48	8 (6.6)
Cavitation (%)*	0 (0)	1 (1.4)	1.0	1 (0.8)
Miliary disease (%)*	0 (0)	1 (1.4)	1.0	1 (0.8)

N/A = not applicable.

* Compared using Fisher’s exact test, 2-tailed.

† Compared using Mann–Whitney *U* test.

Data on symptoms at presentation were recorded for 116 (96%) patients (Table 1). The most common presenting symptoms were fever (*N* = 38), weight loss (*N* = 28), headache (*N* = 26), cough (*N* = 22), local pain or tenderness (*N* = 20), malaise (*N* = 20), appetite loss (*N* = 11), gastrointestinal symptoms (*N* = 10), neck pain (*N* = 10), and dyspnea (*N* = 8). Median duration of symptoms from onset until the lymph node biopsy was 3 months (IQR 1–12 months). HIV-positive patients were more likely than HIV-negative patients to report fever (47% and 24% respectively, *P* < 0.05) and gastrointestinal symptoms (15% and 4.1% respectively, *P* < 0.05).

Chest X-rays were available for 101 patients (84%) and radiological abnormalities were reported for 40 of these patients. Specific abnormalities included pleural effusion(s) (*N* = 12), pulmonary infiltrates and/or consolidation (*N* = 11), hilar and/or paratracheal adenopathy (*N* = 8), cavitation (*N* = 1), and miliary changes (*N* = 1). Radiological abnormalities were present in 30% of HIV-positive and 47% of HIV-negative patients (*P* = 0.087) but there was no association between HIV infection and any specific radiological abnormality (Table 1).

**Figure 1.**
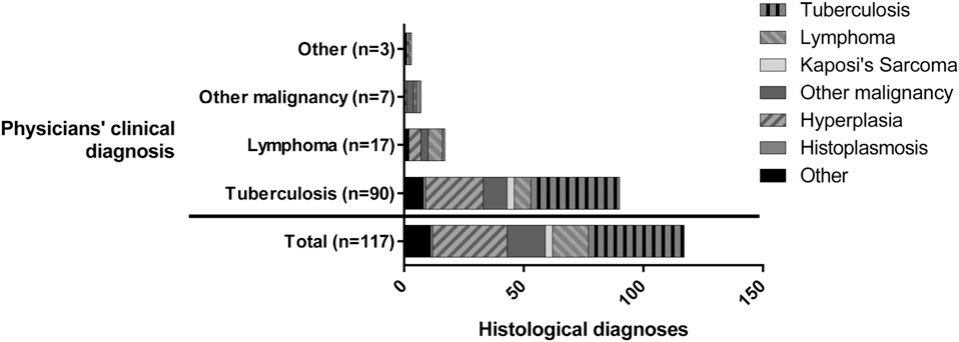
Stacked bar chart showing histological diagnoses where a diagnosis was reached by consensus (Total (*N* = 117), and according to physicians’ clinical diagnosis). The patients’ physicians were asked to give the most likely diagnosis based on available clinical information only. Physicians predicted tuberculosis in the majority of patients (*N* = 90, 77%), lymphoma in 17 patients (15%), other malignancy in seven patients (6.0%), and gave an alternative diagnosis in 3 patients (2.6%). Histological diagnoses included tuberculosis (*N* = 40, 34%), lymphoma (*N* = 15, 13%), Kaposi’s sarcoma (*N* = 3, 2.3%), other malignancy (*N* = 16, 14%), hyperplasia (*N* = 31, 27%), histoplasmosis (*N* = 1, 0.85%), and other diagnoses (*N* = 11, 9.4%). Clinical diagnoses correlated poorly with histological diagnoses.

A histological diagnosis was reached by agreement between ≥ 2 pathologists for 117 patients (97%, Table 2, Figure 1). The most common histological diagnoses were TB (34%, *N* = 40), reactive hyperplasia (27%, *N* = 31), lymphoma (12.8%, *N* = 15), and other malignancy (14%, *N* = 16). Inter-observer agreement between the three pathologists’ diagnoses was high, particularly for TB (κ = 0.85), hyperplasia (κ = 0.64), lymphoma (κ = 0.66), Kaposi’s sarcoma (κ = 0.87), and other malignancy (κ = 0.88). Of the patients who had a diagnosis of “other” (*N* = 11), the tissue sample had been deemed inadequate or non-lymph node in 8 cases.

**Table 2:** Histopathological findings and diagnoses for HIV-positive and HIV-negative patients in whom final diagnosis was reached by consensus by two or more pathologists (*N* = 117)

		HIV positive (%) (*N* = 46)	HIV-negative/status unknown (%) (*N* = 71)	*P* value*	Total (%) (*N* = 117)
Diagnosis of TB	Overall TB diagnosis	16 (35)	31 (44)	0.44	47 (40)
	Histological TB diagnosis	12 (26)	28 (39)	0.17	40 (34)
	Microbiological TB diagnosis	12 (26)	26 (37)	0.31	38 (33)
Histological diagnosis	TB	12 (26)	28 (39)	0.17	40 (34)
	Lymphoma	1 (2.2)	14 (20)	0.005	15 (13)
	KS	2 (4.4)	1 (1.4)	0.56	3 (2.6)
	Other malignancy	3 (6.5)	13 (18)	0.098	16 (14)
	Hyperplasia	24 (52)	7 (9.9)	< 0.001	31 (27)
	Histoplasmosis	1 (2.2)	0 (0)	0.39	1 (0.85)
	Other	3 (6.5)	8 (11)	0.52	11 (9.4)
Histological findings in patients with histological diagnosis of TB	Acid-fast bacilli	2/12 (17)	0/28 (0)	0.085	2 (1.7)
	Granuloma	12/12 (100)	28/28 (100)	1.0	42 (36)
	Caseous material	8/12 (67)	26/28 (93)	0.055	34 (29)

* Compared using Fisher’s exact test, 2-tailed.

Patients with lymphoma were older than patients without lymphoma (median age 62 versus 38 years, *P* = 0.0021) and patients with hyperplasia were more likely to be male than those with other diagnoses (77% versus 49%, *P* = 0.011). Lymph node hyperplasia was more common, and lymphoma less common, among HIV-positive than HIV-negative patients (*P* < 0.001 and *P* = 0.005, respectively). A histological diagnosis of lymphoma was associated with headache, which was reported in 7/15 patients with lymphoma versus 18/102 patients without lymphoma, (*P* < 0.05). Hyperplasia was associated with fever (16/31 patients with, versus 21/86 patients without hyperplasia, *P* = 0.007) and diarrhea (4/31 patients with, versus 2/86 patients without hyperplasia, *P* = 0.042). Patients with lymphoma or other malignancy were less likely to report cough and/or difficulty in breathing than patients with other diagnoses (0/15 pts with, versus 30/102 patients without lymphoma, *P* = 0.011; 8/16 patients with, versus 22/101 patients without non-lymphoma malignancy, *P* = 0.028). There was no association between abnormalities on chest X-ray and histological diagnosis (data not shown).

Granulomata were observed by ≥ 2 pathologists in samples from 42 patients. One of these patients had *Histoplasma capsulatum* detected on both histological analysis and fungal culture (patient HIV positive with a CD4 count of 28 cells/mm^3^), and one patient was assigned a diagnosis of hyperplasia. The remaining 40 patients were given a histological diagnosis of TB. Caseous necrosis was observed in samples from 34 patients; granulomata were also reported in all 34. Inter-observer agreement was high for the presence of granulomas and caseous necrosis (κ = 0.83 and 0.90, respectively), but not AFB (κ = 0.18): AFB were observed on ZN staining by two or more pathologists in just two specimens, both from HIV-positive patients. Caseous necrosis tended to be observed less frequently in samples from patients who were HIV-positive than from those who were HIV-negative (67%, *N* = 46 versus 93%, *N* = 71; *P* = 0.055). Of 40 patients who were assigned a diagnosis of TB according to histological criteria, 31 patients were also positive according to microbiological criteria. Microbiological data have been reported in detail elsewhere.^6^ Eight patients were positive according to microbiological, but not histological, criteria. These patients had histological diagnoses of: hyperplasia (*N* = 4), lymphoma (*N* = 1), other malignancy (*N* = 1), other diagnosis (*N* = 1), and no diagnosis reached by consensus (*N* = 1). Patients who met histological but not microbiological criteria for TB were more likely to have been treated for TB in the past than those who were also positive by microscopy and culture (5/9 patients versus 2/31 patients, respectively, *P* = 0.003).

**Figure 2.**
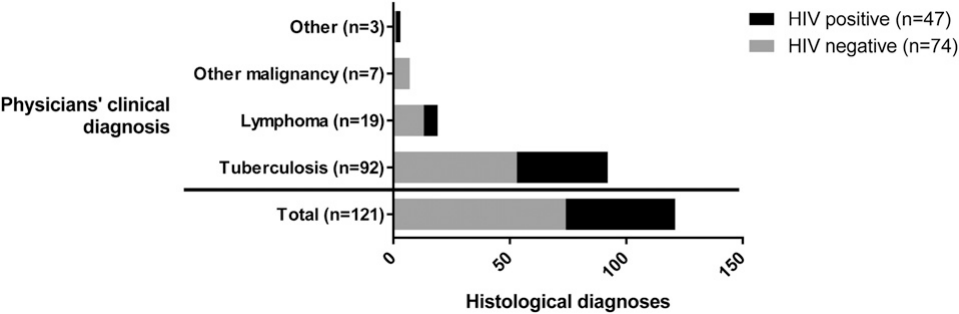
Stacked bar chart showing presumptive clinical diagnosis given by physicians prior to biopsy, according to HIV status. Nonlymphoma malignancy was associated with negative HIV status: it was diagnosed clinically in seven HIV-negative patients and in no HIV-positive patients (*P* = 0.029). HIV status did not affect the frequency of predicted TB (39/47 HIV-positive patients vs 53/74 HIV-negative patients, *P* = 0.19), lymphoma (6/47 HIV-positive patients vs 13/74 HIV-negative patients, *P* = 0.61), or other diagnoses (2/47 HIV-positive patients vs 1/74 HIV-negative patients, *P* = 0.33).

Clinical diagnoses given by the patients’ physicians prior to biopsy did not predict histological diagnosis (Figure 1). The most common clinical diagnosis was TB (*N* = 92, 76%), including for 45/48 patients (94%) who subsequently had a histological and/or microbiological diagnosis of TB and for 47/73 patients (64%) in whom no evidence of TB was found. TB was also the most common predicted diagnosis for patients with all other histological diagnoses. TB, lymphoma, and other malignancy together accounted for the majority of clinical diagnoses (98% of patients), whereas these three conditions comprised less than 50% of histological diagnoses. There was no difference in frequency of clinical diagnosis of TB or lymphoma according to HIV status (Figure 2). A clinical diagnosis of nonlymphomatous malignancy was only given to patients who were HIV negative (7/74 versus 0/47, *P* = 0.029).

## DISCUSSION

This study defined the clinical and histopathological characteristics of a prospective cohort of Peruvian patients with lymphadenopathy. The spectrum of conditions causing lymphadenopathy was broad and comprised a mixture of infective, malignant, and benign causes similar to findings from case series in Peru^8^ and resource-limited settings elsewhere.^9–14^ Clinical characteristics were not specific in attaining a diagnosis. TB was given as the most likely diagnosis in the majority of the patients (76%), whereas in fact hyperplasia, lymphoma, and other malignancy each also contributed a significant burden of disease. These findings highlight the importance of tissue sampling for histological evaluation as part of a thorough diagnostic workup.

Clinical presentation was heterogeneous and there was little relationship between either symptoms or radiological findings and diagnosis, even when disaggregated according to HIV status. Increasing age was associated with malignancy, as in previous reports.^15,16^ Fever and gastrointestinal symptoms were more frequent in HIV positive compared with HIV-negative patients, but the low median CD4 count in our cohort of patients means that such symptoms are likely to be manifestations of advanced HIV infection itself rather than of a coexisting disease.

Interobserver concordance was high with respect to histological features and diagnoses given by the pathologists. Accuracy of histopathological assessment may be dependent on the skill and experience of the observer, but in clinical practice may be greater than that observed, since the pathologist is not usually blinded to clinical information but instead interprets observations within a clinical context. AFB visualization after ZN staining on histopathological analysis was infrequent compared with rates reported in other series.^8,14,17^ AFB detection in fixed tissue specimens is notoriously challenging and time-consuming. Although there have been efforts to devise a classification or grading system for histopathological evaluation of TB lymphadenitis,^18^ interpretation remains variable and in busy clinical settings patterns in tissue architecture such as caseous necrosis may be relied upon more heavily than direct observation of organisms. Granulomata and caseous necrosis are useful to diagnose TB but do also occur in fungal diseases,^19^ which may be missed as a result. It may thus be useful to carry out additional tests, including PAS and/or Silver staining, when this is observed to detect fungal causes.

There was some discrepancy between the histopathological and microbiological diagnoses of TB, with 31 patients positive according to both, nine patients positive according to histological examination only, and eight patients positive according to microbiological criteria only. An exclusively histological diagnosis was associated with previous episodes of TB. It is possible that persistent changes in tissue architecture from previously treated TB may have led to false-positive diagnoses, and/or that recent use of antibiotics with some anti-mycobacterial effect may have led to false-negative microbiological results.^20^

In practice, it may be necessary to initiate treatment of some patients before accurate diagnostic information is available. An over-estimation of the number of patients with TB by their physicians, as we found in our cohort, may lead to empirical anti-TB chemotherapy in a number of patients with other diseases whilst awaiting test results and/or assessment of clinical response to treatment. Although this can lead to adverse effects of unnecessary drugs, this strategy may not be wholly inappropriate as in resource poor settings TB is the most frequent, easily treatable cause of lymphadenopathy.

The trend seen in our data towards reduced frequency of caseous necrosis in samples from HIV positive compared with HIV-negative patients (*P* = 0.055) is contrary to what was expected. In pulmonary TB lesions in HIV-positive patients, poorly formed granulomas with extensive tissue necrosis are frequently seen.^21,22^ It is plausible that HIV-positive patients with severe disease including caseous necrosis may be more likely to be commenced on anti-TB treatment based on the results of other investigations, or empirically on clinical grounds. Such patients would not have undergone lymph node tissue sampling and thus would not have been eligible for this study.

A significant proportion of the patients within this cohort were HIV-positive, with a low median CD4 count of 156 cells/mm^3^. For many patients, this episode of illness was the first presentation of their HIV, reflected in the low proportion of patients already established on antiretroviral therapy (ART) at the time of biopsy (36%). The higher rates of hyperplasia observed amongst HIV-positive than HIV-negative patients may indicate occurrence of the immune reconstitution inflammatory syndrome (IRIS), which frequently involves lymph nodes, in some of the patients taking ART.^23^ In a retrospective meta-analysis, 16% of TB/HIV co-infected patients on ART developed IRIS when TB treatment was commenced, and this was associated with a mortality of 3.2%.^24^ This association of a histological diagnosis of hyperplasia with HIV infection is also likely to account for the increased frequency of fever and diarrhea found to be associated with hyperplasia. A possible explanation for the higher frequency of hyperplasia among HIV-positive patients may be that these patients have an undiagnosed pathology. A postmortem study of 16 HIV-positive patients who died in the Hospital Nacional Dos De Mayo, Lima, Peru, found that 14 (88%) had at least one AIDS-related disease that had not been suspected or diagnosed ante-mortem.^25^

A significant limitation of this study is that patients were not followed up after the biopsy. Some patients may have undergone further investigation before a definitive diagnosis was reached, and/or their initial diagnosis may have been revised. A further limitation is that we did not perform serological testing for other infective causes of lymphadenopathy such as Epstein–Barr virus, cytomegalovirus, toxoplasmosis, brucellosis, or *Coxiella*. It is possible that some of the patients had these investigations performed outside the study. These additional tests were not included in our study due to limited resources, and because the focus of the main study was on TB diagnostics. Therefore, we may have missed some diagnoses, or incorrectly ascribed them to hyperplasia, particularly in the HIV-positive patients.

In summary, this study showed that the spectrum of clinical diagnoses in Peruvian patients with lymphadenopathy is broad, and comprises a mixture of infective, malignant, and benign causes. Clinical manifestations can be highly variable and are not reliable for making a diagnosis. When investigating these patients it is important to perform a full diagnostic workup. This should include tissue sampling for diagnostic testing where resources permit. Histological evaluation may be key in reaching a diagnosis, and should not be delayed. The current approach of treating for TB if histology is not available or is nondiagnostic is pragmatic, but will lead to significant over-treatment with the associated risks of adverse drug reactions.
